# Gut Antibody Deficiency in a Mouse Model of CVID Results in Spontaneous Development of a Gluten-Sensitive Enteropathy

**DOI:** 10.3389/fimmu.2019.02484

**Published:** 2019-10-23

**Authors:** Ahmed Dawood Mohammed, Md. A. Wadud Khan, Ioulia Chatzistamou, Douja Chamseddine, Katie Williams-Kang, Mason Perry, Reilly Enos, Angela Murphy, Gregorio Gomez, Ahmed Aladhami, Carole A. Oskeritzian, Amy Jolly, Yan Chang, Shuqian He, Zui Pan, Jason L. Kubinak

**Affiliations:** ^1^Department of Pathology, Microbiology, and Immunology, University of South Carolina School of Medicine, Columbia, SC, United States; ^2^School of Veterinary Medicine, University of Baghdad, Baghdad, Iraq; ^3^Biology Department, University of Texas at Arlington, Arlington, TX, United States; ^4^Department of Biomedical Sciences, College of Medicine, University of Houston, Houston, TX, United States; ^5^College of Nursing and Health Innovation, University of Texas at Arlington, Arlington, TX, United States

**Keywords:** CVID, primary immunodeficiency, dysbiosis, CVID enteropathy, gluten sensitivity

## Abstract

Primary immunodeficiencies are heritable disorders of immune function. CD19 is a B cell co-receptor important for B cell development, and CD19 deficiency is a known genetic risk factor for a rare form of primary immunodeficiency known as “common variable immunodeficiency” (CVID); an antibody deficiency resulting in low levels of serum IgG and IgA. Enteropathies are commonly observed in CVID patients but the underlying reason for this is undefined. Here, we utilize CD19^−/−^ mice as a model of CVID to test the hypothesis that antibody deficiency negatively impacts gut physiology under steady-state conditions. As anticipated, immune phenotyping experiments demonstrate that CD19^−/−^ mice develop a severe B cell deficiency in gut-associated lymphoid tissues that result in significant reductions to antibody concentrations in the gut lumen. Antibody deficiency was associated with defective anti-commensal IgA responses and the outgrowth of anaerobic bacteria in the gut. Expansion of anaerobic bacteria coincides with the development of a chronic inflammatory condition in the gut of CD19^−/−^ mice that results in an intestinal malabsorption characterized by defects in lipid metabolism and transport. Administration of the antibiotic metronidazole to target anaerobic members of the microbiota rescues mice from disease indicating that intestinal malabsorption is a microbiota-dependent phenomenon. Finally, intestinal malabsorption in CD19^−/−^ mice is a gluten-sensitive enteropathy as exposure to a gluten-free diet also significantly reduces disease severity in CD19^−/−^ mice. Collectively, these results support an effect of antibody deficiency on steady-state gut physiology that compliment emerging data from human studies linking IgA deficiency with non-infectious complications associated with CVID. They also demonstrate that CD19^−/−^ mice are a useful model for studying the role of B cell deficiency and gut dysbiosis on gluten-sensitive enteropathies; a rapidly emerging group of diseases in humans with an unknown etiology.

## Introduction

Primary immunodeficiencies are heritable disorders of immune system function that most commonly manifest as antibody deficiencies (1). Over two dozen genes have been associated with antibody deficiency and many are associated with defects in B cell receptor signaling or co-stimulatory pathways (1). Antibody deficiencies are divided into several categories based on molecular, diagnostic and clinical criteria (2). IgA deficiency is the most common form of antibody deficiency in humans with an incidence of 1 in approximately every 300 people (2–4). IgA deficiency can manifest by itself (selective IgA deficiency) or as part of a more severe antibody deficiency syndrome termed common variable immunodeficiency (CVID). CVID is a primary immunodeficiency disorder characterized by low serum titers of IgG, IgA, and sometimes also IgM (5). In humans, both sIgAD and CVID have been associated with increased susceptibility to infectious, inflammatory, and allergic diseases (4–7).

IgA is by far the most abundantly secreted antibody in the gut and several structural features distinguish secretory IgA from other immunoglobulins as well as from its monomeric form found in serum. Secretory IgA is dimeric due to incorporation of the J-chain during secretion from plasma cells, is heavily glycosylated, and is found complexed with secretory component (SC); a cleavage product of the polymeric Ig receptor (PIgR) that facilitates transcytosis of IgA through gut epithelial cells (8, 9). SC is also heavily glycosylated, and is important for concentrating secretory IgA in the mucus layer covering the luminal surface of the gut epithelium (10). Collectively, these features of secretory IgA position it as a key regulator of interactions between host tissues and the multitude of commensal bacteria that reside in the gut (i.e., the gut microbiota). Secretory IgA is thought to regulate these interactions via three mechanisms; blocking access of bacterial toxins to epithelial cell receptors, immune exclusion (i.e., agglutination), and by regulating virulence factor expression [reviewed in (11, 12)].

CVID patients are acutely susceptible to recurrent pneumococcal infection of the respiratory tract because of a lack of natural serum IgG and IgM antibodies reactive to capsular polysaccharides (5). However, infectious and non-infectious enteropathies are also commonly observed in CVID patients (7), which implies that antibody deficiency also disrupts gut homeostasis and contributes to disease in this patient cohort. Surprisingly, little is currently known regarding how deficits in gut antibody responses contribute to disease in CVID patients, but it has been proposed that gut IgA is important for regulating microbiota composition and pathogenicity. This is supported by multiple mouse models demonstrating that IgA regulates microbiota composition and consequently protects from microbiota-dependent diseases (13). Emerging data from CVID patients now lends further support to the notion that gut antibody deficiency may have a pathological role. For example, in a recent study of a large cohort of CVID patients in Scandanavia, Jørgensen et al. found that CVID patients presenting with (serum) IgA deficiency had altered microbiota composition, enhanced intestinal leakage of microbial products into the bloodstream, and elevated baseline levels of inflammatory serum biomarkers (14). This data set is important because it implies that IgA deficiency could represent an important underlying risk factor for numerous diseases driven by chronic low-grade inflammation in the gut, which challenges the widely held assumption that IgA deficiency is a clinically benign form of antibody deficiency. Importantly, all studies in mouse models to date have relied on chemically-induced or infectious animal models of disease. No study has yet demonstrated an effect of gut antibody deficiency on steady-state physiology in an animal model of CVID.

From a genetic standpoint, CVID is generally considered a polygenic disorder (15–17), however defects in single genes associated with B cell development can also be responsible. CD19 is one of several co-receptors expressed on the surface of B cells that facilitates B cell activation and terminal differentiation into Ig-secreting plasma cells. CD19 deficiency is a known genetic risk factor for the development of monogenic CVID in humans (1), and CD19^−/−^ mice present with very similar humoral defects to those observed in human CVID patients (defective B cell development, terminal differentiation into isotype-switched plasma cells, and low serum titers of IgG, IgA and IgM) (18). CD81 and CD21 also form part of the CD19 co-receptor complex and defects in these genes are also associated with CVID in humans (2). CD20 is also associated with CVID in humans, but in mice does not appear to generate as severe a humoral immune deficiency as that seen in CD20-deficient humans (19). Here, we utilize CD19^−/−^ mice as a model of monogenic CVID arising due to a BCR co-receptor defect to test the hypothesis that gut antibody deficiency negatively impacts gut physiology by promoting chronic inflammation under steady-state conditions. Results from our experiments show that CD19^−/−^ mice develop a deficiency in gut antibody production that is associated with altered microbiota composition characterized by outgrowth of anaerobic bacteria. More importantly, this is associated with the spontaneous development of an intestinal malabsorption disorder driven by chronic inflammatory responses that appear to be localized to the small intestine of mice. As a consequence, these mice suffer from multiple deficiencies associated with nutrient (primarily lipid) transport and metabolism. Furthermore, antibiotic treatment and exposure to a gluten-free diet rescue CD19^−/−^ mice from malabsorption indicating involvement of both the microbiota and dietary gluten exposure in pathogenesis.

## Materials and Methods

### Mouse Models, Husbandry, and Diet

WT C57BL/6 and CD19^−/−^ mice (also C57BL/6 background) were bred and maintained at the University of South Carolina Animal Care Facility. To initially generate this colony, WT C57BL/6 (Jax#000664) and CD19^−/−^ (Jax#006785) mice were purchased from Jackson Laboratories. WT and CD19^−/−^ mice were crossed to generate F_1_ heterozygotes and were subsequently crossed to re-derive homozygote breeding lines. All animals used in the experiments described here are derived from this colony. Animals were reared and maintained under identical SPF conditions in a single environmentally-controlled room exclusively used to house this mouse colony. Experimental data were derived from mixed groups of male and female mice between the ages of 8 and 16 weeks old. Animals are maintained under constant environmental conditions (70°F, 50% relative humidity, 12:12 light:dark cycles) and were given *ad libitum* access to autoclaved drinking water and an irradiated soy-free mouse chow that utilizes gluten as the dominant protein source (Envigo; diet#2920X; 19% crude protein). For antibiotic exposure experiments, animals were weaned at 3 weeks of age and given *ad libitum* access to autoclaved drinking water containing 0.5 mg/mL of metronidazole for 5 weeks. Antibiotic water was replaced weekly and stool samples were collected for weekly enumeration of fecal anaerobic CFUs. To test whether malabsorption is a gluten-sensitive enteropathy in CD19^−/−^ mice, animals were exposed at 3 weeks of age until 8 weeks of age to a gluten-free mouse chow that incorporated casein (rather than gluten) as the dominant protein source (Bioserv; diet#F1515-AIN-76A; 18% crude protein). To avoid GFD diet contamination animals were maintained in autoclaved cages and housed and handled separately from animals on standard chow. All animal use strictly adhered to federal regulations and guidelines set forth by the University of South Carolina Institutional Animal Care and Use Committee (Protocol#101292).

### Lymphocyte Isolations

*Peritoneal lavage cell isolation*: The peritoneum of euthanized mice were exposed with scissors and a 10 cc syringe was used to fill the peritoneal cavity of animals with 5 mL of sterile 1X HBSS buffer. The body cavity was then gently massaged to dislodge cells. Peritoneal lavage fluids were drawn back into the same syringe and placed in a 15 mL conical tube on ice for downstream processing. *Peyer's patch (PP) cell isolation:* PPs were collected from animals and a single cell suspension was made by passing cells through a 40 μm cell strainer. Cells were washed once in complete RPMI media (RPMI 1640 supplemented with FBS, sodium pyruvate, non-essential amino acids, L-glutamine, penicillin-streptomycin, and β-ME) and collected for analysis. *Lamina propria cell isolation*: Lymphocytes from small intestine or colon lamina propria were isolated via enzymatic digestion of gut tissues as previously described (20).

### Fecal Short-Chain Fatty Acid (SCFA) Quantification

Fresh fecal pellets were collected from WT and CD19^−/−^ mice by having animals defecate directly into sterile 1.5 mL microfuge tubes. Processing of fecal samples and quantification via gas chromatography using a flame iodization detector (GC-FID) was conducted as described previously (21). Briefly, fecal samples were weighed and subsequently homogenized in 1 mL of 0.5% phosphoric acid solution using a Bead-beater (Biospec). Prior to homogenization, 2-ethyl butyric acid was added to the solution to serve as an internal standard. The homogenate was centrifuged at 13,000 RPM for 10 min. Five-hundred microliters of methyl tert-butyl ether (MTBE) was added to the resulting supernatant. The samples were then vortexed, centrifuged for 10 min at 13,000 RPM, and the supernatant was isolated for downstream analysis. Analysis of SCFAs was performed on an HP5890 Series II gas chromatograph (Agilent) equipped with a standard flame ionization detector. Two microliters of the MTBE solution was injected in splitless mode (splitless time 30 s). The GC column was a 30 meter Stabilwax-DA with 0.25 mm inner diameter and 0.25 μm film thickness (Resek). The carrier gas was helium at 15 psi. The oven temperature started at 100°C and ramped immediately (i.e., hold time zero) at 7°C/min to 250°C (hold time 5 min). Injection port was 250°C and FID was 275°C.

### Flow Cytometry

All flow cytometry was carried out on a FACS Aria II instrument (BD). Primary cells derived from PPs, peritoneal lavage, or LP isolations were washed once in ice cold 1X PBS and then re-suspended in 100 μL volumes of staining buffer (1XHBSS + 5 mL FBS + 5 mL 0.5 M EDTA) containing antibodies against cell surface markers to discriminate the following cell subsets: (PP follicular T_FH_ cell; CD4^+^CD3ε^+^PD1^+^CXCR5^+^), (PP germinal center B cells B220^+^IgDlowGL7^+^FAS^+^), (PEC B2 cells; B220^+^CD43^−^CD5^−^CD4^−^), (PEC B1a cells; B220^+^CD43^+^CD5^+^CD4^−^), (PEC B1b cells; B220^+^CD43^+^CD5^−^CD4^−^), (LP IgA^+^ Plasmablasts; B220^−^CD138^+^IgA^+^). Specific information on the antibodies used in this study is provided in Supplementary Table S1. For a detailed description of gating strategies used to enumerate cell subsets please refer to Supplementary Figure S2.

### Quantitative PCR and Quantitative RT-PCR

qPCR was used to quantify bacterial abundance in feces and mucosal scrapings. For mucosal scrapings, the gut tube (small and large intestine; cecum excluded) was splayed open with scissors in a sterile petri dish and loose fecal matter was rinsed from tissues with sterile 1X PBS. Using sterile forceps (re-sterilized by wiping down, rinsing in 70% EtOH, and heating with flame in between samples) the entire mucosal surface was scraped and materials were placed in a bead-beater tube for downstream DNA isolation. DNA from fecal pellets and mucosal scrapings was isolated using the Powerfecal DNA isolation kit (with 3 min bead-beating step) (Qiagen). DNA concentration was measured using a Nanodrop spectrophotometer. The relative abundance of bacterial 16S rRNA gene copies was enumerated on a 7300 qPCR instrument (Applied Biosystems) using the PowerUp SYBR Green Master kit and universal bacterial primers. Total copies of 16S rRNA gene copies were standardized to the amount of input DNA per reaction. For gene expression experiments, ileal tissues (1 cm proximal to cecum) were collected and placed in 1.5 mL microfuge tubes containing 250 μL RNAlater solution (Ambion) and frozen at −80°C until downstream RNA isolation. RNA was extracted from tissues using an RNeasy kit (Qiagen). DNase I digestion (Sigma) was used to remove contaminating DNA from RNA preparations and was subsequently converted to cDNA using the qScript cDNA Synthesis Kit (Quanta). The iTaq Universal SYBR Green Master Mix (Bio-Rad) was used for qRT-PCR reactions and reactions were run on a CFX96 Real-time PCR System (Bio-Rad). Gene expression data was normalized to GAPDH or β-actin housekeeping genes. Primer sets used in this study are as follows: [universal bacteria forward-334F: 5′- ACTCCTACGGGAGGCAGCAGT-3′; universal bacteria reverse-514R: 5′- ATTACCGCGGCTGCTGGC (22)]; [tgm2 forward: 5′-TTCCGGCTGACTCTGTACTTCGAG-3′; tgm2 reverse: 5′-ACATTGTCCTGTTGGTCCAGCACT-3′ (23)]; (Cpa3 forward: 5′- TCAGACCATCCAGTCAACCTT-3′; Cpa3 reverse: 5′-CCTGCCTGCGATTTCATCTTT-3′); (GAPDH forward: 5′-ATGGTGAAGGTCGGTGTGAAC-3′; GAPDH reverse: 5′-GCCTTGACTGTGCCGTTGAAT-3′); β-actin forward: 5′-GACGGCCAGGTCATCACTATTG-3′; (β-actin reverse: 5′-AGGAAGGCTGGAAAAGAGCC-3′).

### 16S rRNA Gene Profiling

Fecal pellets and ileal contents were collected from animals and placed directly into 2 mL collection tubes containing sterile garnet stones for downstream bead-beating and DNA isolation following the Powerfecal DNA Isolation Kit instructions (Qiagen). Briefly, mice were scruffed and allowed to defecate directly into a collection tube. For ileal contents, from euthanized mice, the ileum was removed with sterile forceps and scissors, and the ileal contents were squeezed directly into a collection tube. All DNA extractions were carried out in a biosafety cabinet. Animals were chosen for inclusion in sequencing experiments using the following criteria: (1) both male and female mice were included for each genotype, (2) animals were derived from multiple breeding cages from each genotype, (3) mice were sampled from several different stock cages for each genotype, (4) mice were derived from different litters from the same breeder pair (5) the age-range of mice (eight to 16 weeks old) was similar between genotypes. Isolated DNA was submitted to the University of Alabama Heflin Center Genomics Core for paired-end 16S sequencing on an Illumina MiSeq instrument. Raw fastq reads were de-multiplexed and forward and reverse primer sequences were trimmed from reads (Trimmomatic). This yielded a 251 bp product spanning the V3/V4 region of the bacterial 16S rRNA gene. De-multiplexed fastq files were imported as QIIME 2.0 artifacts for downstream processing and analysis using the QIIME 2.0 analysis pipeline (24). De-multiplexed reads were trimmed (forward 250 bp; reverse 200 bp) based on quality score cutoff of Q <15. Denoising was performed using DADA2 with amplicon sequence variant (ASV) taxonomic calls being made against the GreenGenes database (13.8). Low quality reads and chimeras were filtered out of final feature table using DADA2. Prior to β-diversity and α-diversity analyses, feature tables were rarified to even sampling depth (fecal community experiment = 41,235 sequences per sample, ileal community experiment = 10,716 sequences per sample, antibiotics experiment = 41,235 sequences per sample) (Supplementary Figure S1A). Based on our analysis, the effect of cage, age, or sex does not explain observed differences in microbiota composition between genotypes (Supplementary Figures S1B–E).

### RNAseq Analysis of Ileal Transcriptomes

Total RNA was extracted from ileum tissue using an RNeasy Mini Kit (Qiagen). Briefly, ileum tissues were disrupted/homogenized in 2-Mercaptoethanol-containing RLT buffer using a TissueLyzer LT (Qiagen) with 7-mm stainless steel beads for 5 min at 50 Hz. The lysate was transferred to a new tube, centrifuged at full speed for 3 min and supernatant was carefully transferred to a new tube. Equal volume of 70% ethanol was added, mixed by pipetting, transferred to a RNeasy spin column and spun down for 15 s at 15,000 g. The column was washed by centrifugation at RT for 15 s at 15,000 g with RW1 buffer, treated with DNase (Qiagen) for 15 min at RT, washed with RW1 buffer, washed with RPE buffer twice, washed with 80% ethanol, and dried by centrifugation for 4 min at full speed. RNA was eluted into a new tube by adding 35 μl of RNase-free water to the column and spinning it for 1 min at 15,000 g. RNA purity and concentration was assessed using a NanoDrop 2000c spectrophotometer (Thermo Scientific). RNA integrity was determined using an Agilent 2100 Bioanalyzer (Agilent) where RNA Integrity Numbers ranged from 9.3 to 9.6 for all samples. RNA libraries were prepared using established protocol with NEBNExt Ultra II Directional Library Prep Kit (NEB, Lynn, MA). Each library was made with one of the TruSeq barcode index sequences and Illumina sequencing was performed by GENEWIZ, Inc. (South Plainfield, NJ) with Illumina HiSeq4000 (150 bp, pair-ended). Sequences were aligned to the *Mus musculus* genome GRCm38.p5 (GCA_000001635.7, ensemble release-88) using STAR v2.4. Samtools (v1.2) was used to convert aligned sam files to bam files and reads were counted using the featureCounts function of the Subreads package with Gencode.vM19.basic.annotation.gtf annotation file. Only reads that were mapped uniquely to the genome were used for gene expression analysis with on average 87 ± 0.01% of all reads being assigned. On average 4% of reads were unassigned due to ambiguous base calling and 9% were unassigned due to non-gene alignment against the mouse genome. Differential expression analysis was performed in R using the edgeR package. The average read depth for the samples was 14,144,535 and only genes with at least one count per million average depth were considered for differential expression analysis. Raw counts were normalized using the Trimmed Mean of M-values (TMM) method. The normalized read counts were fitted to a quasi-likelihood negative binomial generalized log-linear model using the function glmQLFit.

### Diarrhea Scoring

Fecal pellets were collected from mice and weighed. The frequency of defection was measured by placing an animal in a cage for 1 min and counting the number of pellets excreted per minute. Fecal water content was measured by weighing fresh fecal pellets, then dehydrating them by heating them at 95°C for 24 h on a thermal block. Pellets were then weighed and the difference between fresh pellet weight and dry pellet weight was quantified. Fecal water weight scores represent this measurement.

### Fecal CFU Titers

Fecal and liver CFUs were enumerated by plating on BHI agar. Livers and feces from animals were weighed and then homogenized in sterile 1X HBSS buffer. Three serial 10-fold dilutions of homogenates were plated on BHI agar plates and incubated for 24 h under either aerobic or anaerobic conditions. CFU counts were standardized by fecal weight.

### Fecal IgA, IgG, IgM, and Serum IgA, IgG, and AGA ELISAs

Fecal antibody titers were measured using isotype-specific Ready-Set-Go ELISA kits (Affymetrix). Fecal pellets were collected and weighed. Pellets were homogenized with a mortar and pestle in sterile 1X HBSS buffer and then spun at 400 × g for 5 min to precipitate course materials. Supernatants were aliquoted into a second 1.5 mL microfuge tube and spun at 8,000 × g for 10 min to precipitate bacteria. Supernatants were collected, placed in a new tube, and spun a second time at 8,000 × g for 10 min. These supernatants were then used for ELISA assays. Antibody concentrations were standardized by fecal weight. Serum measurement of AGA antibodies was done using the Mouse Anti-Gliadin Antibody (AGA) ELISA Kit (MYBioSource). Serum measurement of IgA and IgA done using the same isotype-specific Ready-Set-Go ELISA kits (Affymetrix) mentioned above.

### IgA-Bound Bacteria Assay

The percentage of IgA-bound bacteria in animals was enumerated via flow cytometry as previously described (20). Briefly, fecal pellets were obtained from animals and homogenized in 1X PBS. Bacterial pellets were stained with a rat anti-mouse-IgA antibody (1:100) conjugated to an APC fluorophore (Southern Biotech) in the dark and on ice for 30 min. Pellets were washed twice in cold 1X PBS and stained with SYBR green (1:10,000; Molecular Probes) to identify bacteria. IgA^+^ bacteria were enumerated by gating on APC^+^SYBR^+^ events. RAG1^−/−^ bacteria were stained as above and used as controls to quantify non-specific binding. The average non-specific staining value from RAG1^−/−^ animals is provided as dashed line in Figure 2A to illustrate the limit of assay detection.

### Histology

Approximately 5 cm of the distal ileum (proximally from the ileo-cecal valve) were collected from euthanized animals and placed immediately in 10% buffered formalin. Tissues were submitted for processing to the USC Instrumentation Resources Facility Histology Core. Paraffin block sections were stained with hematoxylin and eosin (H&E) and subsequently analyzed blindly by a certified pathologist and characterized according to the presence of histo-morphological changes of inflammation. For the evaluation of the specimens of the distal ileum the scoring system suggested by Erben et al. (25) was applied and the parameters evaluated included inflammatory infiltrate (severity and extent), as well as epithelial (crypt hyperplasia) and mucosal (villous blunting) changes of the intestinal wall. At least 4–5 consecutive villi from base to tip were rated. Tissue sections were examined and images were obtained by standard light microscopy using a Leica optical microscope system. The entire colon was removed from WT and CD19^−/−^ mice and placed in 10% buffered formalin. Colons were sectioned and H&E stained. Colon inflammation was scored using a previously established scoring system for inflammatory bowel disease in mice (26). Anti-CD3 immunohistochemistry staining was performed by the USC Instrumentation Resources Facility Histology Core using a polyclonal rabbit anti-mouse-CD3 antibody (ABCAM, ab5690). For high resolution representative images for animal cohorts described in this study please refer to Supplementary Figure S4.

### FITC-Dextran Gut Permeability Assay

Animals were fasted for 8 h with *ad libitum* access to autoclaved drinking water. Animals were then orally gavaged with FITC-dextran (3,000–5,000 MW; Sigma) at a dose of 60 mg/100 g body weight. After gavage, animals were housed without food (but given *ad libitum* access to drinking water) for an additional 4 h and were then euthanized with CO_2_ per Federal and USC IACUC guidelines. Whole blood was collected from euthanized animals via cardiac puncture. Serum FITC-dextran concentrations were measured using a Synergy 2 fluorescence plate reader (BioTek) with a laser excitation of 485 nm and detection emission wavelength of 528 nm.

### Statistical Analysis

**Figure 1:**
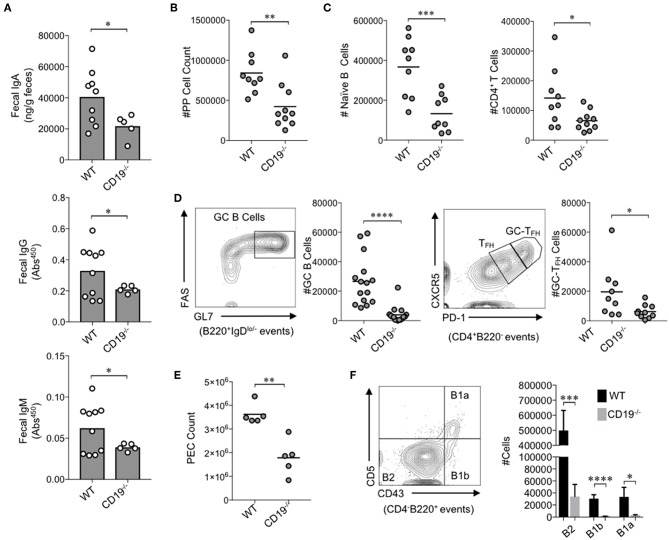
CD19^−/−^ mice develop gut mucosal antibody deficiency due to defects in B cell development in gut-associated lymphoid tissues. **(A)** The fecal concentrations of IgA, IgG, and IgM are shown. **(B)** The total number of lymphocytes isolated from PPs of mice is shown. **(C)** The total number of naïve B cells and CD4^+^ T cells in PPs is shown. **(D)** A representative flow plot demonstrating gating on GC B cells and GC-T_FH_ cells is provided and the total abundance of GC B cells and GC-T_FH_ cells is shown. **(E)** The total number of peritoneal lavage cells is shown. **(F)** A representative flow plot demonstrating gating on peritoneal B cell subsets is provided and the total abundance of B2, B1a, and B1b cells is shown. **(A–F)** Student's *t*-test (* = *p* < 0.05, ** = *p* < 0.01, *** = *p* < 0.001, **** = *p* < 0.00001). See Supplementary Figure S2 for details on gating strategy.

**Figure 2:**
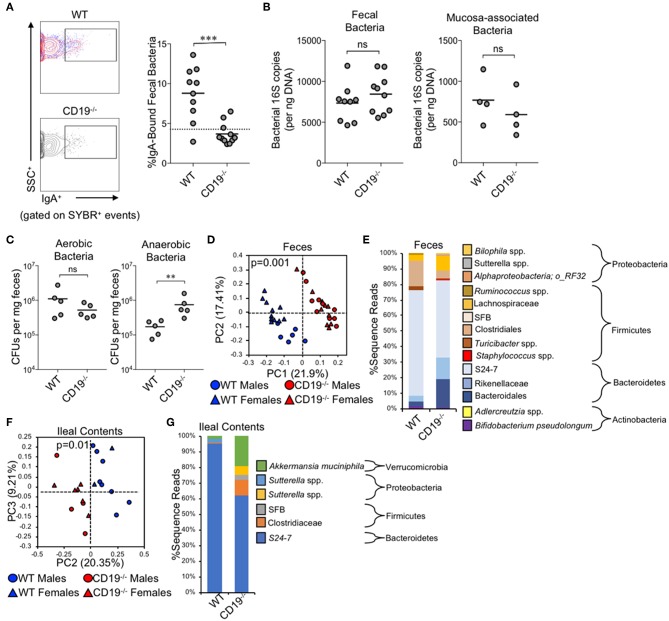
CD19^−/−^ mice bind less commensal bacteria with IgA and have outgrowth of anaerobic bacteria. **(A)** A representative flow cytometry plot of IgA-bound bacteria gating is provided and the percentage of bacteria coated with IgA is shown. **(B)** The total abundance of fecal and mucosa-associated (small and large intestine) bacteria were quantified by qPCR. **(C)** The abundance of fecal anaerobic and aerobic CFUs were enumerated. **(D)** PcoA plot based on unweighted UniFrac analysis of β-diversity between WT and CD19^−/−^ fecal bacterial communities. **(E)** Stacked barplot summarizing fecal bacterial groups whose abundance is significantly altered in CD19^−/−^ mice. **(F)** PcoA plot based on Bray-Curtis analysis of β-diversity between WT and CD19^−/−^ ileal bacterial communities. **(G)** Stacked barplot summarizing ileal bacterial groups whose abundance is significantly altered in CD19^−/−^ mice. **(A–C)** Student's *t*-test; ns, non-significant, ** = *p* < 0.01, *** = *p* < 0.001. **(D,F)** *P*-value represents result of PERMANOVA testing with 999 permutations. **(E,G)** Significant groups of differentially enriched bacteria were identified with discrete FDR testing.

**Figure 3:**
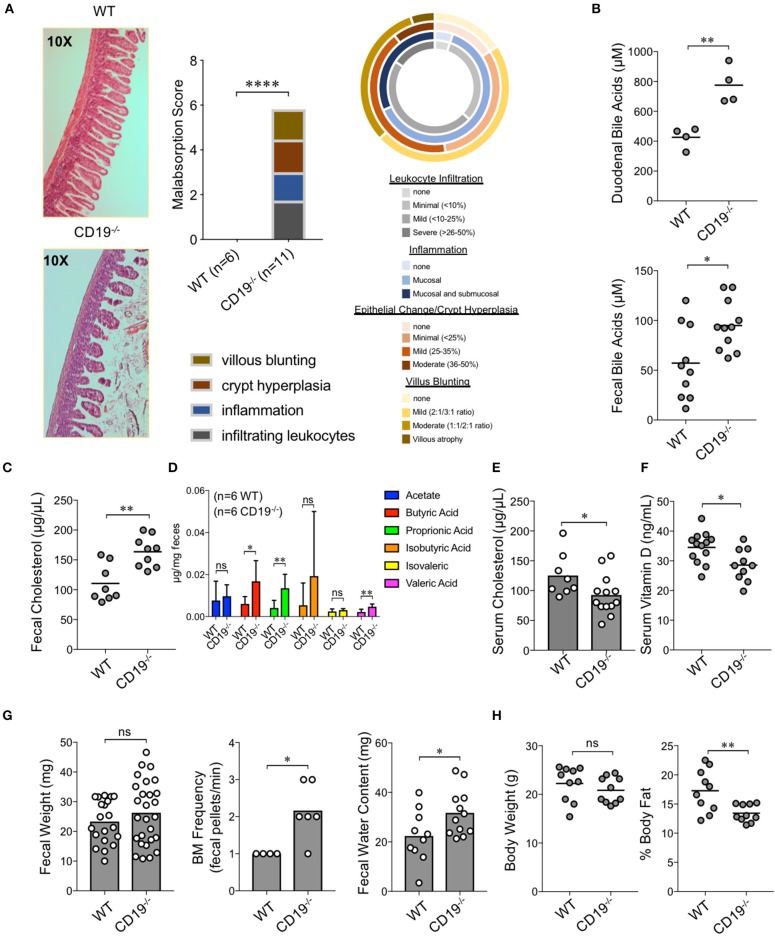
Gut IgA deficiency is associated with intestinal malabsorption in CD19^−/−^ mice. **(A)** H&E histological analysis of ileum from WT and CD19^−/−^ mice using a 4-point malabsorption scoring index. Circle plot represents the percentage of CD19^−/−^ animals falling into the respective score grouping. Colors represent severity (darker means more severe and the size of colors per circle represent percentage of animals in that category). **(B)** Total duodenal and fecal BA concentrations are shown. **(C)** Total fecal cholesterol levels are shown. **(D)** Fecal SCFA concentrations are shown. **(E)** Serum cholesterol titers are shown. **(F)** Serum vitamin D titers are shown. **(G)** Fecal weight, defecation frequency, and fecal water content are shown. **(H)** Body weight and results of DEXA scan of fat mass are shown. **(B–G)** Student's *t*-test; * = *p* < 0.05, ** = *p* < 0.01. Error bars in D represent S.D. **(A)** Result of Two-way ANOVA (row factor = genotype), **** = *p* < 0.0001.

Data sets for all experiments represent pooled data from two to three experimental replicates (*n* = 2–3 animals per replicate). Unless otherwise noted in figure captions, a Student's *t*-test was used for significance testing. Error bars in Figures 1F, 3D, 5A,D represent S.D. For statistical analysis of 16S data a PERMANOVA (999 iterations) was used to test whether β-diversity is significantly different between genotypes. Stacked barplots in Figures 2E,G, 5F summarize bacterial groups whose abundance is significantly different between genotypes or treatments. These bacterial groups were identified using the discrete FDR (DS-FDR) analysis method integrated into the QIIME 2.0 analysis pipeline. For RNAseq data, gene-wise statistical tests for significant differential expression were conducted with empirical Bayes quasi-likelihood F-tests using the function glmQLFTest. Significant differentially expressed genes were included in analysis that had an FDR *q*-value of < 0.2. In Figures 3A, 6B, 7C, a two-way ANOVA was used as a statistical test to compare malabsorption severity between genotypes. *P*-values are results of row-factor comparison (row factor = genotype).

## Results

### B Cell Deficiency in Gut-Associated Lymphoid Tissues (GALT) of CD19^−/−^ Mice Results in Gut Antibody Deficiency

We first wanted to know whether the antibody deficiency previously characterized in the systemic compartment of CD19^−/−^ mice also manifested as an antibody deficiency in the gut. To test this, we measured fecal IgA, IgG, and IgM titers in WT and CD19^−/−^ mice. The fecal concentrations of IgA, IgG, and IgM were all significantly reduced in CD19^−/−^ mice compared to WT controls (Figure 1A). IgA is the most abundantly secreted antibody in the gut with very low titers of IgG and IgM being detected in our mice [above background but below the limit of the sensitivity of standards (<1 ng)]. CD19^−/−^ mice demonstrated a two-fold reduction in IgA compared to WT mice (average 20.2 vs. 40.5 μg/g, respectively). Thus, IgA deficiency is by far the most severe antibody deficit in the gut of CD19^−/−^ mice. To more fully define this deficiency, we compared relevant immune cell subsets within the GALT [i.e., the small intestinal Peyer's patches (PPs)] via flow cytometry. The total cellularity of PPs in CD19^−/−^ mice was significantly reduced in comparison to WT controls (Figure 1B). Flow cytometric analysis revealed that most of this reduction in PP cellularity is due to significant contraction of the PP B cell population, but T cell abundance is also reduced (Figure 1C). Qualitative differences in PPs cell subsets were also observed. Specifically, germinal center (GC) B-cell (B220^+^IgD^lo−^GL7^+^FAS^+^) development was significantly reduced in CD19^−/−^ mice as was the abundance of GC-T_FH_ cells (CD4^+^B220^−^CXCR5^hi^PD-1^hi^) (Figure 1D). Thus, CD19^−/−^ mice have a severe deficit in their ability to mount T-cell-dependent antibody responses to luminal antigens. In addition to B cells of the GALT, peritoneal B cell subsets [particularly the B-1 subsets; B-1a (CD4^−^B220^+^CD5^+^CD43^+^); B-1b (CD4^−^B220^+^CD5^−^Cd43^+^)] are also important for generating T-cell-independent anti-commensal IgA responses in the gut (27). As reported previously (18), we confirm that CD19^−/−^ mice have significantly fewer cells in peritoneal lavage fluids compared to WT mice (Figure 1E), which was associated with significant reductions in the abundance of all B cell subsets (B2, B1a, B1b) (Figure 1F). We next compared the abundance of IgA^+^ plasmablasts (CD138^+^B220^+^IgA^+^) in the effector sites of the gut [i.e., the small intestine lamina propria (SiLP) and colonic lamina propria (CLP)]. Interestingly, CD19^−/−^ mice had significantly reduced abundance of IgA^+^ plasmablasts in the SiLP but not the CLP, indicating a possible site-specific defect in the recruitment of plasmablasts to the small intestine (Supplementary Figure S2C).

### CD19^−/−^ Mice Cannot Bind Commensal Bacteria With IgA and This Is Associated With Expansion of Fecal Anaerobic Bacteria

Given the antibody deficiency and defects observed in the immune cell subsets relevant to T-cell-dependent and T-cell-independent anti-commensal IgA responses, we next wanted to determine whether this translated into defects in IgA-binding of commensal microbes. Using a flow cytometry approach, we found that CD19^−/−^ mice bound a significantly lower fraction of commensal microbes with IgA than WT animals (3.4% in CD19^−/−^ mice vs. 8.8% in WT mice) (Figure 2A). In fact, IgA-binding of bacteria was below the limit of assay detection for most CD19^−/−^ mice, which we deduced by comparing the degree of non-specific staining observed when microbes derived from RAG1^−/−^ mice were stained with the same anti-mouse-IgA antibody (dashed line in Figure 2A). We next wanted to determine if an inability to bind commensal bacteria with IgA led to gross differences in the total abundance or composition of the microbiota in CD19^−/−^ mice. Quantitative PCR analysis of bacterial 16S rRNA gene copy abundance from feces or mucosal scrapings from the small and large intestine (minus cecum) did not reveal any differences between WT and CD19^−/−^ mice in total bacterial abundance within these materials (Figure 2B). However, we did observe qualitative differences in the relative abundance of aerobic and anaerobic bacteria. CD19^−/−^ mice had an overgrowth of anaerobic bacteria in their feces compared to WT animals, while aerobic CFU titers were equivalent between these genotypes (Figure 2C). To validate these observations and more fully describe compositional differences in the microbiota we performed 16S rRNA gene sequencing on both fecal and ileal bacterial communities in WT and CD19^−/−^ mice. Fecal (Figure 2D; unweighted UniFrac) and ileal (Figure 2F; unweighted UniFrac) community composition (i.e., β-diversity) was significantly different in CD19^−/−^ mice compared to WT animals. We did not observe any differences between genotypes when comparing a variety of α-diversity metrics (Supplementary Figure S3). In both communities, we found significant outgrowth of multiple groups of obligate and facultative anaerobic bacteria. Notably, in the feces of CD19^−/−^ mice we observe outgrowth of numerous species within the order Bacteroidales, the families Rikenellaceae and Lachnospiraceae, as well as specific species; segmented filamentous bacteria (SFB), *Staphylococcus* spp., *Sutterella* spp., an undescribed species of alphaproteobacteria, and *Bilophila* spp. (Figure 2E). In the ileum we find outgrowth of members of the family Clostridiaceae, as well as specific species; SFB, *Sutterella* spp, and *Akkermansia muciniphila* (Figure 2G). See Supplementary Tables S2, S3 for full list of differentially abundant ASVs in fecal and ileal materials, respectively. Collectively, these results indicate that defective anti-commensal IgA responses correlate with compositional shifts in the microbiota and the effect is dominated by outgrowth of anaerobic bacteria in the gut.

### CD19^−/−^ Mice Develop Chronic Intestinal Malabsorption

To determine whether gut antibody deficiency and altered microbiota composition influenced gut inflammation we performed blinded histological analysis of colon and ileal tissues taken from WT and CD19^−/−^ mice. While we did not see any evidence of inflammation in the colons of CD19^−/−^ mice (Supplementary Figure S5), we observed several abnormalities in the small intestine. Specifically, in contrast to WT mice, we found that CD19^−/−^ mice had villus blunting, crypt hyperplasia, inflammation, and lymphocyte infiltration in their ileum (Figure 3A). Collectively, these are hallmark features of intestinal malabsorption. Since inflammation increases gut permeability (28), we sought to determine if the histological defects resulted in a leaky-gut syndrome. Therefore, we measured serum concentrations of a passively absorbed fluorescent dye (FITC-dextran) 12 h after oral gavage of this reagent into WT and CD19^−/−^ mice. Results of our experiments demonstrated that CD19^−/−^ mice had increased gut permeability compared to WT animals under steady-state conditions (Supplementary Figure S6). As further validation of a malabsorption disorder, we assessed fecal and serum pool sizes of relevant molecules. The ileum is the primary site of bile acid absorption in the gut. Bile acid concentrations in both duodenal contents and fecal pellets were significantly elevated in CD19^−/−^ mice, consistent with bile acid malabsorption (Figure 3B). Bile acids facilitate lipid absorption (primarily cholesterol) in the ileum and we found that fecal cholesterol levels are significantly elevated in CD19^−/−^ mice (Figure 3C). Additionally, multiple fecal short-chain fatty acids that are produced by the microbiota were also significantly elevated in CD19^−/−^ mice (Figure 3D). In contrast, serum cholesterol levels were reduced (Figure 3E). We also found evidence that absorption of lipid-soluble vitamins was perturbed in CD19^−/−^ animals as serum vitamin D levels were significantly reduced in these animals (Figure 3F). CD19^−/−^ mice also developed a mild diarrhea characterized by increased fecal output rate and fecal water content (Figure 3G). Finally, X-ray analysis of body composition demonstrated that CD19^−/−^ mice, while having a similar body weight and lean mass compared to WT mice, had significantly reduced body fat mass (Figure 3H). Collectively, these data demonstrate that CD19^−/−^ mice suffer from a chronic lipid malabsorption disorder.

### Ileal Transcriptomics Identifies Chronic Inflammatory Immune Responses as the Driver of Intestinal Malabsorption

**Figure 4:**
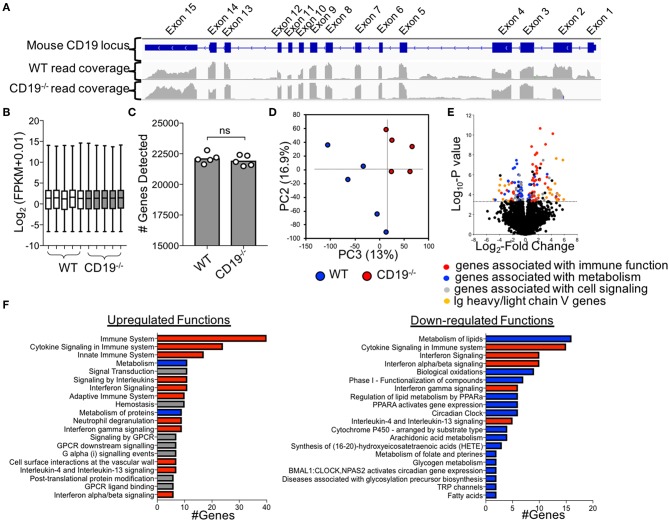
Comparison of Ileal Transcriptomes of WT and CD19^−/−^ mice. **(A)** Read mapping across CD19 locus in WT (*n* = 5) and CD19^−/−^ (*n* = 5) mice. **(B)** FPKM values of normalized RNAseq data. **(C)** The number of genes whose transcripts were identified in RNAseq. **(D)** PC plot of ileal transcriptomes of WT and CD19^−/−^ mice demonstrating clustering by genotype. **(E)** Volcano plot with differentially enriched (DE) genes highlighted by function. Dotted line represents significance cutoff; FDR < 0.2. **(F)** Pathway (i.e., Reactome) analysis of upregulated and downregulated functions in CD19^−/−^ mice compared to WT controls. **(F)** Red bars indicate functions associated with immune response, blue bars indicate functions associated with metabolism, gray bars indicate other functions.

To more fully define the malabsorption phenotype in CD19^−/−^ mice and to identify possible factors driving disease, we performed RNAseq to compare the ileal transcriptomes of WT and CD19^−/−^ mice. Cre-transgene insertion into the first exon of the CD19 locus abolishes CD19 function (18). Read-mapping at the CD19 locus confirmed this deletion in CD19^−/−^ mice (Figure 4A). RNAseq analysis of transcripts was normalized to fragment per kilobase of transcripts per million (FPKM) values (Figure 4B) and an equivalent number of genes were detected between genotypes (Figure 4C). The ileal transcriptome of CD19^−/−^ mice diverged significantly from WT animals (Figure 4D). The most apparent difference in ileal gene expression in CD19^−/−^ mice compared to WT mice was a clear inverse relationship between the expression of inflammatory immune genes and metabolic genes (Figure 4E). Pathway analysis identified several metabolic pathways downregulated in the ileum of CD19^−/−^ mice including lipid metabolism, biological oxidations, and Phase I functionalization of compounds (Figure 4F). In contrast, upregulated genes were primarily associated with aspects of innate and adaptive immune responses (Figure 4F) (A full list of differentially expressed genes is provided in Supplementary Table S4).

### Immune Signature of Intestinal Malabsorption in CD19^−/−^ Mice Is Dominated by Mast Cell and T Cell Responses

**Figure 5:**
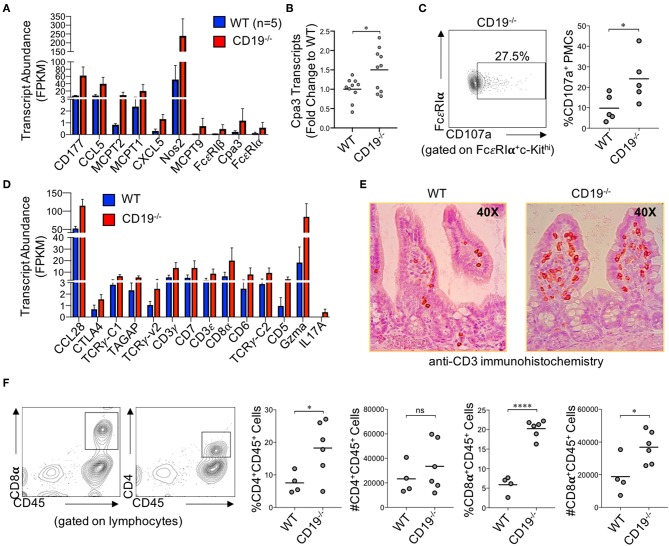
Immune Involvement in Intestinal Malabsorption in CD19^−/−^. **(A)** DE gene transcripts in CD19^−/−^ mice associated with innate immunity. **(B)** qRT-PCR of Cpa3 mRNA expression. **(C)** Representative flow cytometry plot of activated peritoneal mast cells with relative abundance shown. **(D)** DE gene transcripts in CD19^−/−^ mice associated with adaptive immunity. **(E)** Representative anti-CD3 immunohistochemistry staining of WT and CD19^−/−^ ileal sections. **(F)** The relative and absolute abundance of CD4 and CD8 T cells isolated from the gut mucosa (lamina propria) of WT and CD19^−/−^ are provided. **(A,D)** Quasi-likelihood ratio test to identify significantly DE genes. **(B,C,F)** Student's *t*-test; ns, non-significant, * = *p* < 0.05, **** = *p* < 0.0001.

Numerous genes associated with innate and adaptive immune responses were upregulated in the ileum of CD19^−/−^ mice. Specifically, multiple genes associated with mast cells were upregulated in CD19^−/−^ mice (Figure 5A). We validated by qPCR that Cpa3 expression, a specific marker of mast cells, was upregulated in the ileum of CD19^−/−^ mice (Figure 5B). Using flow cytometry, we also confirm that CD19^−/−^ mice have a higher percentage of activated peritoneal mast cells (FcεR1α^+^cKit^+^CD107^+^ cells) (29) (Figure 5C) (Supplementary Figure S7). The majority of upregulated immune genes were associated with T cell responses (Figure 5D). We performed two assays to determine if T cells were increased in the gut mucosa of CD19^−/−^ mice. First, we performed anti-CD3 immunohistochemistry staining to visualize mucosal T cells in the ileum of WT and CD19^−/−^ mice. Results from anti-CD3 IHC staining supported RNAseq data by demonstrating more T cells within the ileum of CD19^−/−^ mice (Figure 5E). Finally, we isolated mucosally-associated T cells from the ileal lamina propria and enumerated CD4 (CD4^+^CD45^+^) and CD8 (CD8α^+^CD45^+^) T cell subsets by flow cytometry. Both the percentage of CD4 and CD8 T cells were significantly increased in the gut of CD19^−/−^ mice, while only the absolute abundance of CD8 T cells were significantly increased (Figure 5F). Collectively, these data indicate that mast cell and mucosal T cells are likely to be the primary immune cells contributing to mucosal inflammation observed in CD19^−/−^ mice.

### Metronidazole Treatment Abolishes Intestinal Malabsorption in CD19^−/−^ Mice

**Figure 6:**
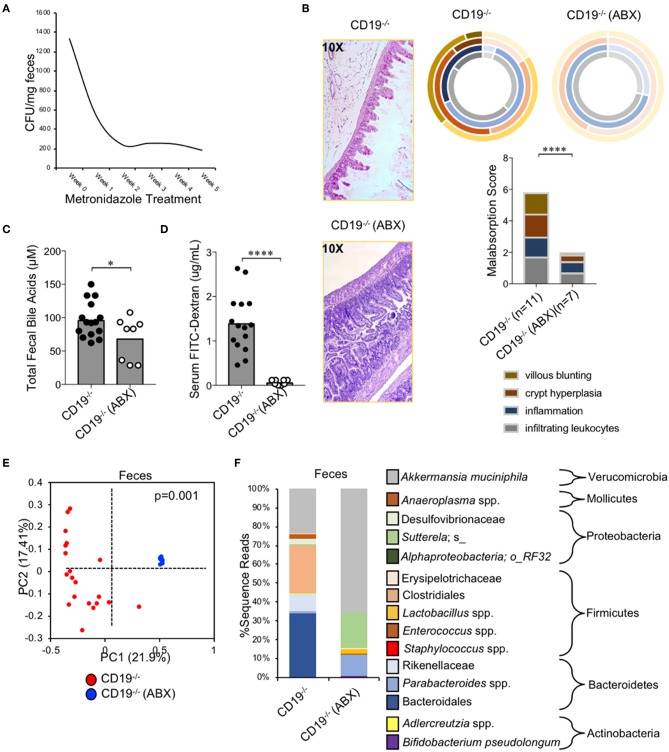
Malabsorption in CD19^−/−^ mice is microbiota-dependent. **(A)** Fecal anaerobic bacteria abundance during 5 week metronidazole exposure. **(B)** Malabsorption scoring for ABX-treated CD19^−/−^ mice compared to untreated controls. **(C)** Total fecal BA quantification of ABX-treated mice vs. controls is shown. **(D)** Serum FITC-Dextran concentrations of ABX-treated mice vs. controls is shown. **(E)** PcoA plot based on unweighted UniFrac analysis of β-diversity between fecal microbial communities from ABX-treated CD19^−/−^ mice compared to untreated controls. **(F)** Stacked barplot summarizing bacterial groups whose abundance significantly changes in ABX-treated mice compared to controls. **(C,D)** Student's *t*-test; * = *p* < 0.05, **** = *p* < 0.0001. **(E)** *P*-value represents result of PERMANOVA testing with 999 permutations. **(F)** Significant groups of differentially enriched bacteria were identified with discrete FDR testing. **(B)** Result of Two-way ANOVA (row factor = genotype), **** = *p* < 0.0001.

To determine if expansion of anaerobic bacteria was driving the malabsorption phenotype observed in CD19^−/−^ mice we depleted anaerobic bacteria via administration of metronidazole (a bacteriostatic antibiotic with selective activity against anaerobic bacteria) in cohorts of CD19^−/−^ mice. Animals were reared on drinking water containing metronidazole for 5 weeks (from weaning age of 3 weeks to 8 weeks of age) and then malabsorption, fecal bile acid output, and gut permeability were compared to that of untreated CD19^−/−^ mice. Metronidazole treatment significantly reduced the abundance of fecal anaerobic bacteria in CD19^−/−^ mice (Figure 6A), and at 8 weeks of age this coincided with significant reductions in the severity of malabsorption (Figure 6B). Metronidazole-treated animals also had reduced fecal BA output (Figure 5C) as well as reduced intestinal permeability (Figure 6D). 16S microbiota profiling of metronidazole-treated mice confirmed a significant shift in microbiota composition due to antibiotic treatment (Figure 6E) with pronounced loss of anaerobes being the dominant feature of this shift. Anaerobic bacterial groups whose abundance was reduced include numerous species with the orders Bacteroidales and Clostridiales, members of the families Rikenellaceae and Desulfovibrionaceae, as well as specific species; SFB, *Staphylococcus* spp., *Sutterella* spp., a species of alphaproteobacteria, and *Bilophila* spp. (Figure 5F). In contrast, metronidazole treatment resulted in the expansion of *Bifidobacterium pseudolongum, Lactobacillus* spp., and *Akkermansia muciniphila* in feces (Figure 6F). A full list of bacteria impacted by metronidazole treatment is provided in Supplementary Table S5.

### Malabsorption in CD19^−/−^ Mice Is a Gluten-Sensitive Enteropathy

**Figure 7:**
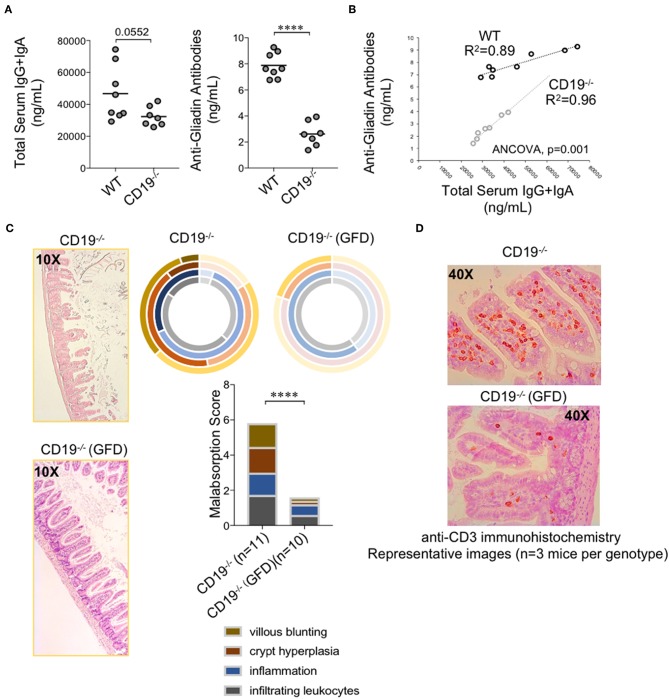
Malabsorption in CD19^−/−^ mice is gluten-sensitive. **(A)** Total serum IgG and IgA and total AGA antibody titers are shown. **(B)** Correlation between AGA antibody titers and total IgG/IgA titers in WT and CD19^−/−^ mice. **(C)** Malabsorption scoring for GFD-treated CD19^−/−^ mice compared to animals reared on a gluten-containing mouse chow. **(D)** Representative anti-CD3 IHC staining of ileal T cells in CD19^−/−^ mice treated with GFD vs. controls. **(A)** Student's t-test, **** = *p* < 0.0001. **(C)** Result of Two-way ANOVA (row factor = genotype), **** = *p* < 0.0001.

Intestinal malabsorption has been linked to enhanced gluten sensitivity; most commonly in the context of celiac disease in humans. This led us to speculate that malabsorption observed in CD19^−/−^ mice may be a gluten-sensitive enteropathy. Gliadin is the major immunogenic component of gluten. Serum antibody testing to measure the abundance of anti-gliadin antibodies (AGAs) is one method for assessing gluten-sensitivity in humans. Given their antibody deficiency, we did not anticipate that CD19^−/−^ mice would produce more total antibodies against gluten antigens. We instead hypothesized that they may have a higher “relative sensitivity” to gluten. To measure “relative sensitivity” we measured the concentrations of anti-gliadin antibodies (AGAs) in mice and correlated these values with total serum antibody titers measured in the same animals. A higher “relative sensitivity” would be indicated by a stronger correlation between AGAs and total serum antibody titers in one genotype over another. The AGA kit utilized measures serum IgG and IgA AGAs so we compared these values with the summed total of serum IgG and IgA titers. As expected, CD19^−/−^ animals had lower total serum IgG and IgA antibodies (Figure 7A; left panel) as well as AGA antibodies (Figure 7A; right panel). However, we observed a significantly stronger positive association between AGA antibody production and total serum IgG and IgA titers in CD19^−/−^ mice (ANCOVA, p<0.001) which we interpret to mean that CD19^−/−^ mice are more responsive to dietary gluten antigens (or food antigens in general) despite their antibody deficiency (Figure 7B). All of the mice in this study were maintained on a standardized mouse chow that includes wheat gluten as the major source of dietary protein. Therefore, to determine if the intestinal malabsorption observed in our CD19^−/−^ mice may be due to dietary gluten exposure we modeled a strict gluten-free diet (GFD) by weaning 3-week-old CD19^−/−^ mice onto a modified gluten-free mouse chow. This chow substitutes casein for gluten as the dominant dietary protein source. Five weeks after initial GFD-exposure we sacrificed mice and measured malabsorption histologically. Results from our experiments indicate that exposure to a GFD rescues the malabsorption phenotype in CD19^−/−^ mice to a similar degree that metronidazole treatment does (Figure 7C vs. **6B**). Exposure to GFD also reduced the number of mucosally-associated T cells observed in the ileum of CD19^−/−^ mice by immunohistochemistry (Figure 7D). These data indicate that intestinal malabsorption in CD19^−/−^ mice is a gluten-sensitive enteropathy.

## Discussion

In a seminal study, Rickert et al. identified CD19 as an important molecule regulating steady-state humoral immune responses and serum antibody titers (18). Since then, this mouse model has been instrumental in furthering our mechanistic understanding of complement-driven B cell activation (30–32), the mechanisms governing BCR activation thresholds (33, 34) and B cell development (35), and the influence of CD19-deficiency in experimental models of infectious (35), autoimmune (31, 36, 37), and inflammatory disease (38, 39). However, no study has used this model to address whether there are spontaneous defects in gut physiology associated with gut antibody deficiency under homeostatic conditions. This is an important question to address because CD19 deficiency is a genetic risk factor for the development of CVID in humans (2, 40, 41) and CVID is associated with a variety of gastrointestinal disorders (7) but the underlying factors driving these associations are unknown. Mouse models are essential to studying this. Here, we present data that complements this previous work by demonstrating that antibody deficiency in CD19^−/−^ mice extends to the gut mucosal compartment. More importantly, results from our experiments are one of the first to clearly define a chronic physiological effect of gut antibody deficiency; intestinal malabsorption. We have been careful to characterize malabsorption several ways including comparing ileal transcriptomes of WT and CD19^−/−^ mice, through histological analysis of ileal tissues, and functionally by demonstrating abnormal BA, lipid, and vitamin concentrations in feces and serum. Finally, we also demonstrate that intestinal malabsorption in CD19^−/−^ mice is a microbiota-dependent and gluten-sensitive enteropathy.

Mutations in several different immune genes associated with B cell development can lead to CVID in humans (2), but the common defining feature of all of these defects is a significant reduction in circulating antibody titers (always IgG in association with low IgA and also sometimes IgM). Here we chose to use CD19^−/−^ mice as a model of CVID because CD19-deficiency is a known monogenic risk factor for CVID (1) and because we wanted to study the effect of antibody deficiency in the context of an intact T cell compartment. In humans, the majority of CVID patients have normal circulating numbers of B cells and CVID seems to arise as a result of defects in terminal B cell differentiation into plasma cells as indicated by the absence of isotype-switched memory B cells in CVID patients (42). CD19^−/−^ mice seem to have a more severe defect in B cell development as that observed in humans because they have significant reductions in all B cells. Regardless, their overall phenotype is similar to that of CVID patients in terms of antibody concentrations in serum and gut secretions. CD19^−/−^ mice also have enhanced recruitment of T cells to the gut suggesting that T cells play an important role in the intestinal malabsorption we find in these animals. This finding is important because (as discussed below) a common feature to all gluten-sensitive enteropathies is increased activation of T cell responses in the gut. We demonstrate that CD19^−/−^ mice (consistent with studies on the systemic compartment of these mice) have developmental abnormalities in the both the B2 and B1 B cell compartments relevant to anti-commensal antibody responses. Both B cell subsets are important for barrier integrity by contributing to the luminal IgA pool. Whether the absence of B1 vs. B2 cells from the gut are more important for controlling abberrant T cell activation and ensuing intestinal malabsorption is not currently known. We believe that much of the aberrant T cell response associated with B cell deficiency arises due to enhanced exposure of T cells in the gut to luminal antigens. However, B cells may also dampen immune responses through the secretion of IL10 (so-called regulatory B cells) and both splenic B cells and B cells from the peritoneal cavity have been shown to control inflammatory T cell responses in chemically-induced models of colitis though this pathway (38, 43, 44). Therefore, we cannot rule out that absence of regulatory B cells also contributes to intestinal malabsorption in CD19^−/−^ mice, though it should be pointed out that no one has yet to report the spontaneous development of intestinal inflammation in B cell-specific IL10-deficient mice despite them having been around for a decade.

To our knowledge, this is the first study to demonstrate that B cell deficiency results in histological defects in the gut epithelium of mice. One potential reason why previous studies have not been able to identify an effect of antibody deficiency on steady-state gut physiology is compensatory immune responses that mask the effect. Enhanced secretion of IgM is one such compensatory response that has been observed in alternative mouse models of antibody deficiency. For example, Aicda^−/−^ mice have a defect in the ability of their B cells to class switch to IgA resulting in an IgA deficiency, but this results in enhanced IgM secretion (45, 46). Similarly, B-cell-intrinsic TGFβRII^−/−^ mice develop a severe IgA deficiency in serum and secretions, but develop more IgM^+^ B cells and demonstrate significantly enhanced antigen-specific IgM production during immunization experiments (47, 48). Indeed, even IgA^−/−^ mice, which represent the most direct genetic manipulation of IgA production, have been reported to develop a hyper-IgM syndrome in serum and mucosal secretions (49), and have also been shown to have a severe defect in IgG production in a model of allergic airway disease (50). A hyper-IgM-like syndrome may also be observed in a subset of CVID and agammaglubulinemia patients (51). No compensatory gut antibody responses are detected in CD19^−/−^ mice and we believe a physiological effect of antibody deficiency can be detected in these mice because of this. Interestingly, results from our experiments closely match observations made by Polly Matzinger's lab who previously used two B cell deficient mouse models (μMT^−/−^ and ${\text{J}}_{\text{H}}^{-/-}$) to study the role of B cell deficiency in regulating gut metabolism (52). Through a series of elegant experiments, these authors were able to demonstrate that loss of protective antibody responses in the gut led to upregulation of immune genes (primarily genes associated with interferon responses) and down-regulation of a number of genes associated with lipid metabolism in the jejunum. We see a very similar pattern of gene expression in the ileum of CD19^−/−^ mice (i.e., immunity at the cost of metabolism), which supports these authors conclusions that lipid malabsorption observed in CVID patients may result from a physiological tradeoff between the gut epitheliums need to regulate its' own immunity at the expense of its metabolic function under conditions of antibody deficiency. However, despite a genetic signature of malabsorption, these authors were unable to detect any of the classic histological features of malabsorption in the jejunum of their animals, whereas we do see histological features of malabsorption in the ileums of CD19^−/−^ mice. This discrepancy could be due to differences in the microbial communities between animal facilities that drive a more severe malabsorption in our animals, or due to differences in where malabsorption presents histologically in a mouse compared to humans. More recently, another study by Shulzhenko et al. compared transcriptomic profiles from duodenal biopsies collected from CVID patients presenting with an enteropathy (E-CVID patients) (defined as chronic diarrhea or unexplained weight loss coupled with clinical signs of protein loss syndrome) vs. CVID patients without enteropathy (noE-CVID patients). Consistent with results from our mouse model and the mouse models and CVID patients described by the Matzinger group, these authors again found that upregulation of immunity genes in E-CVID patients was inversely correlated with lipid metabolism genes, whereas this was not observed in noE-CVID patients (53). Collectively, results from our work and others support that defects in lipid absorption may be either a consequence or contributing factor to the enhanced inflammatory responses observed in CVID-enteropathy. Finally, while our data suggests that IgA deficiency is responsible for intestinal malabsorption, we cannot conclusively say this without directly manipulating the IgG, IgA, and IgM responses in the gut. However, in the same study mentioned above, the Matzinger Group used AID^−/−^/μs^−/−^ mice, which possess B cells but cannot secrete antibodies, to show that these mice have a similar phenotype to B cell deficient μMT^−/−^ and ${\text{J}}_{{\text{H}}^{-/-}}$ mice (52). This provides strong support for the argument that antibody-deficiency, rather than loss of other potential B cell functions (e.g., immunosuppression), is likely to be the main driver of observed defects in gut metabolism in B cell deficient animals. We are now developing models to directly manipulate gut antibody responses in an isotype-specific manner in CD19^−/−^ mice.

A 40 year longitudinal analysis of 473 CVID patients found that gastrointestinal inflammatory disease and malabsorption explained ~21% of all CVID-associated complications (54). The underlying factors driving this effect are still undefined. The gastrointestinal tract represents the largest mucosal surface of the human body. IgA concentrates in the mucus layer (55) and is therefore positioned to provide first-line defense against bacterial adherence to the gut epithelium and bacterial translocation into the systemic compartment (11, 12). An earlier study by Perreau et al. found that CVID patients had higher serum endotoxin titers suggesting that bacteria, or their products, were translocating from the gut into the systemic compartment (56). This was subsequently also observed in a Scandinavian cohort of CVID patients, and was also correlated with reduced serum IgA levels and altered microbiota composition (14). Interestingly, and more recent, Romberg et al. also observed elevated serum endotoxin titers in CVID patients; specifically in a subset of CVID patients with associated autoimmune-induced cytopenias (CVID+AIC patients) (57). These researchers hypothesized that cytopenia was due to an inability of these patients to limit mucosal bacterial penetration from the gut leading to activation of autoreactive B cell clones. Moreover, CVID+AIC patients also had significantly reduced IgA^+^ memory B cells and significantly lower serum IgA titers compared to CVID patients without AIC. Thus, accumulating evidence suggests that CVID-associated IgA deficiency disrupts normal host-microbiota interactions and drives disease. To what degree shifts in microbiota composition, an inability to manage bacterial translocation from the gut, or altered host metabolism drives disease in CVID patients is not known. Collectively, these studies imply that IgA deficiency results in defects in the integrity of the gut epithelium. Here, we demonstrate that antibody deficient CD19^−/−^ mice develop increased intestinal permeability and malabsorption driven by inflammatory immune responses directed against the microbiota as well as dietary antigens like gluten. To our knowledge, the phenotype we have described in CD19^−/−^ mice mirrors many of the non-infectious complications commonly associated with CVID in humans. Thus, we believe this model will be valuable for future studies seeking to empirically dissect the association between gut dysbiosis and CVID. Finally, we would like to point out that under the highly controlled environment of an SPF facility, CD19^−/−^ mice are viable and do not present with any discernible physiological defects. Similarly, despite emerging evidence to the contrary, IgA deficiency in humans is considered a benign form of antibody deficiency due to the general absence of clinical symptoms. One important conclusion from our study is that gut antibody deficiency results in chronic low-grade inflammation that has an important physiological impact on host metabolism. Thus, the cumulative effect of damage to the gut epithelium or chronic immune activation could be a predisposing factor to the development of multiple diseases later in life.

The question arises of what drives the chronic inflammation that appears to be localized to the small intestine of CD19^−/−^ mice. We believe three factors could contribute to this site-specific effect; the inherently higher permeability of this tissue, the outgrowth of anaerobic bacteria and elevated bile acid concentrations. Our metronidazole experiments demonstrate that malabsorption is a microbiota-dependent phenomenon associated with expansion of anaerobes. The observed expansion of anaerobic bacteria in mice is similar to a human condition termed “small intestinal bacterial overgrowth” (SIBO). SIBO is characterized as a pathological overgrowth of anaerobic bacteria in the human small intestine (58). SIBO is commonly observed in CVID patients (7) and is also strongly associated with malabsorption and celiac disease (58, 59). Results from our experiments parallel observations in human SIBO patients. First, antibiotic treatment has been shown to resolve inflammation and malabsorption in elderly SIBO patients (60), and metronidazole is more effective than other antibiotics at treating SIBO and resolving its associated inflammatory conditions (61, 62). These observations are important because they demonstrate that the microbiota plays a prominent role in malabsorption associated with intestinal bacterial overgrowth, and that outgrowth of anaerobic bacteria may be a principle microbial driver of inflammation. Results from our antibiotic experiment only say that anaerobic bacteria, which are expanded in our mice, have a proinflammatory role that contributes to intestinal malabsorption. We cannot currently determine whether the presence or absence of specific anaerobes, their overall enhanced abundance or metabolic contribution to the biochemical environment of the gut, or the commensurate reduction in abundance of beneficial microbes drives intestinal malabsorption in our model. Indeed, this is an extremely difficult question to answer and is beyond the scope of the current study. Current efforts in our lab are now focused on understanding how the microbiota alters the biochemistry of the gut in CD19^−/−^ mice to drive disease. For example, the microbiota are key players in bile acid homeostasis in the gut where bacterial deconjugation of bile acids is the rate-limiting step in secondary bile acid synthesis (63). Due to the potential cytotoxicity of bile acids, their synthesis and recycling in the gut is a strictly controlled process under normal physiological conditions. Our results demonstrate that bile acid concentrations are significantly elevated in the small and large intestines of CD19^−/−^ mice and this could by itself contribute to malabsorption by enhancing apoptosis of gut epithelial cells. Additionally, the microbiota formed in CD19^−/−^ mice could further enhance the cytotoxic effects of bile acids by altering the composition of the bile acid pool in the gut. Ongoing experiments in our lab are exploring these questions. Finally, in the same study mentioned above, Shulzhenko et al. addressed the question of whether CVID-enteropathy was associated with differences in the microbiome between E-CVID and noE-CVID patients. These authors identified subtle differences in microbiota composition among these patient cohorts. Specifically, this group observed that mucosal IgA deficiency, which was a universal feature in E-CVID patients but not noE-CVID patients, was strongly linked to outgrowth of the pathobiont *Acinetobacter baumanni*. This group then went on to show that the genetic signature of CVID enteropathy could be recapitulated *in vitro* using an intestinal cell line co-cultured with *A. baumanni*. This study supports that specific members of the microbiota can drive intestinal malabsorption.

Results from our experiments indicate that intestinal malabsorption in CD19^−/−^ mice is a gluten-sensitive enteropathy. Broadly, gluten sensitivity is defined as any disease whose symptoms positively respond to the removal of gluten from the diet (64). There are several forms of gluten sensitivity that can be broadly distinguished by the quality of the immune response that develops in response to gluten antigen. Wheat allergy involves allergic immune responses driven by release of mediators from basophils and mast cells, celiac disease which is characterized as an autoimmunity do the generation of self-reactive autoantibodies, and non-celiac gluten sensitivity that strongly resembles celiac disease but does not involve an autoimmune response to self antigens (65). All three forms of gluten sensitivity involve activation of T cells in the gut mucosa. Celiac disease is the most well-characterized gluten-sensitive enteropathy and intestinal malabsorption is a common feature of this disease (66–68). An immune reaction to dietary gluten is thought to be the primary driver of celiac disease, and 90–95% of all celiac disease patients carry at least one copy of specific HLA-DQ alleles (DQ2/DQ8) that enhances an individual's ability to present modified gluten peptides via MHC class II molecules to T cells (69). However, a subset of celiac disease patients will have unresolved symptoms even with strict adherence to a GFD (70) and only 3% of all carriers of these HLA risk alleles will develop celiac disease (71). Thus, in addition to genetic predisposition and dietary exposure to gluten, there are additional environmental factors contributing to this disease that have not yet been identified. Celiac disease has been linked to abnormal gut microbiota composition (72). Furthermore, it has been proposed that secretory IgA promotes the establishment of a “healthy” microbiota (13), and selective IgA deficiency is 10–15 times more prevalent in celiac disease patients (73–77). Therefore, selective IgA deficiency may promote celiac disease progression by driving dysbiosis that enhances gluten sensitivity. Along with IgA deficiency, CVID has also been associated with a specific form of celiac disease termed “seronegative celiac disease (SNCD)” (77). SNCD is characterized by absence of autoreactive serum antibodies and primary antibody deficiencies are one potential explanation for sero-negativity in this subset of celiac patients. This is an important point to consider, because it highlights how an underlying immunodeficiency can make a diagnosis of celiac more difficult. How the antibody deficiency in CD19^−/−^ mice contributes to gluten sensitivity and what specific form of gluten sensitivity CD19^−/−^ mice develop is currently unknown but our data suggest that the microbiota could be an important sensitizing factor. Interestingly, one possible mechanism by which microbes may sensitize the immune system to gluten is through modification of gluten antigens by microbial transglutaminase. This enzyme appears to mimic the function of host tissue transglutaminase in its ability to deamidate and enhance the immunogenicity of gluten peptides (78). Additionally, it's also possible that microbial molecules may simply have an adjuvant effect on T cells in the gut exacerbating a response to innocuous food antigens. Future co-housing experiments between WT and CD19^−/−^ mice, or microbiota transplantation studies into germfree WT mice will be useful for determining if the unique microbiota composition formed in CD19^−/−^ mice is sufficient to drive gluten sensitivity and intestinal malabsorption. There are two caveats that we would like to point out in regards to the interpretation of our data. Our results indicate that intestinal malabsorption in CD19^−/−^ mice is a gluten-sensitive enteropathy associated with enhanced T cell responses in the gut. However, we cannot deduce from these studies whether it is driven by a gluten-specific T-cell response, or whether it is simply a general reaction to food antigens (where gluten simply represents the dominant protein antigen encountered). We would also like to acknowledge that the GFD used in this study was not completely nutritionally matched to the gluten-containing mouse chow so it is possible that other nutritional differences could play a role in the protective effect observed in our mice. However, given the fact that simply eliminating gluten from the diet leads to complete remission of gut inflammation in the vast majority of celiac patients (that are otherwise eating highly diversified diets) we would conclude that the effect is driven by the absence of dietary gluten. Moreover, even with the use of a completely nutritionally-matched diet, lipid malabsorption in CD19^−/−^ mice profoundly alters the biochemical environment of the gut making it impossible to say that gluten antigens alone are the specific driver of inflammation (e.g., enhanced lipid concentrations in the gut of CD19^−/−^ mice could increase the production of proinflammatory lipid mediators like arachidonic acid). Experiments are underway in our lab to address these points. Despite these limitations, we believe that the CD19^−/−^ mouse will be useful for future research on how gut dysbiosis driven by primary antibody deficiency influences gluten-sensitive enteropathies; a rapidly emerging group of related diseases in humans with a currently undefined etiology.

## Data Availability Statement

All datasets generated for this manuscript are provided in the manuscript or supplementary materials. Raw 16S fastq files will be made available upon request.

## Ethics Statement

The animal study was reviewed and approved by University of South Carolina Institutional Animal Care and Use Committee.

## Author Contributions

AM conducted all experiments shown and assisted in manuscript writing. MK conducted experiments and assisted in manuscript writing. IC performed all histological analyses of ileal samples. DC, KW-K, and MP performed bacterial CFU enumeration and qPCR of bacteria. RE and AM were involved in bacterial SCFA measurements and DEXA scanning of animals. GG and CO provided advice and assistance with mast cell work. GG assisted in flow cytometric analysis of peritoneal mast cells. CO and AA performed qPCR of Cpa3 gene. ZP, YC, and SH performed blinded colon inflammation scoring. AJ performed all mouse husbandry and ran fecal ELISAs to quantify antibody titers. JK conceived of all experiments, oversaw all aspects of the work detailed here, and wrote the first and final draft of this manuscript.

### Conflict of Interest

The authors declare that the research was conducted in the absence of any commercial or financial relationships that could be construed as a potential conflict of interest.

## Supplementary Material

Supplementary Table 1 List of antibodies used.

Supplementary Table 2 Differentially enriched fecal OTUs.

Supplementary Table 3 DE Ileal OTUs.

Supplementary Table 4 Differentially enriched ileal transcripts.

Supplementary Table 5 Differentially enriched OTUs in metronidazole-treated mice.
