# Supplementary material for: Gut Antibody Deficiency in a Mouse Model of CVID Results in Spontaneous Development of a Gluten-Sensitive Enteropathy
Source: Front Immunol. 2019 Oct 23;10:2484. doi: 10.3389/fimmu.2019.02484 (PMC6820504; doi:10.3389/fimmu.2019.02484)
Supplement: Supplementary Table 1 — List of antibodies used. [file Table_1.pdf]

Supplementary Table 1. List of flow cytometry antibodies used in this study

Antibody	Fluor	Clone	Vendor	Catalog #	Concentration
Anti-mouse CD4	FITC	GK1.5	Biolegend	100406	0.5mg/ml
Anti-mouse CD19	PercP.Cy5.5	1D3/CD19	Biolegend	152405	0.2mg/ml
Anti-mouse B220	PercP.Cy5.5	RA3-6B2	Biolegend	103235	0.2mg/ml
Anti-mouse GL7	FITC	GL7	Biolegend	144612	0.5mg/ml
Anti-mouse FAS	PE	SA367H8	Biolegend	152607	0.2mg/ml
Anti-mouse CD43	APC	S11	Biolegend	143207 143208	0.2mg/ml
Anti-mouse CD5	PE	53-7.3	Biolegend	100607	0.2mg/ml
Anti-mouse CD138	APC	281-2	Biolegend	142505	0.2mg/ml
Anti-mouse CD3ε	APC.Cy7	145-2C11	Biolegend	100330	0.2mg/ml
Anti-mouse PD-1	PE.Cy7	29F.1A12	Biolegend	135215	0.2mg/ml
Anti-mouse CXCR5	PE	L138D7	Biolegend	145503	0.2mg/ml
Anti-mouse IgA	PE	11-44-2	Southern Biotech	1165-09L	0.1mg/ml
Anti-mouse CD8a	PE	53-6.7	Biolegend	100708	0.2mg/ml
Anti-mouse cKit	APC	2B8	Biolegend	105811	0.2mg/ml
Anti-mouse CD107a	PE	1D4B	Biolegend	121611	0.2mg/ml
Anti-mouse CD45	PE.Cy7	30-F11	Biolegend	103113	0.2mg/ml
Anti-mouse FcεR1α	FITC	MAR-1	Biolegend	134305	0.5mg/ml
