# Supplementary material for: Gut Antibody Deficiency in a Mouse Model of CVID Results in Spontaneous Development of a Gluten-Sensitive Enteropathy
Source: Front Immunol. 2019 Oct 23;10:2484. doi: 10.3389/fimmu.2019.02484 (PMC6820504; doi:10.3389/fimmu.2019.02484)
Supplement: Supplementary file 6 [file Data_Sheet_1.pdf]

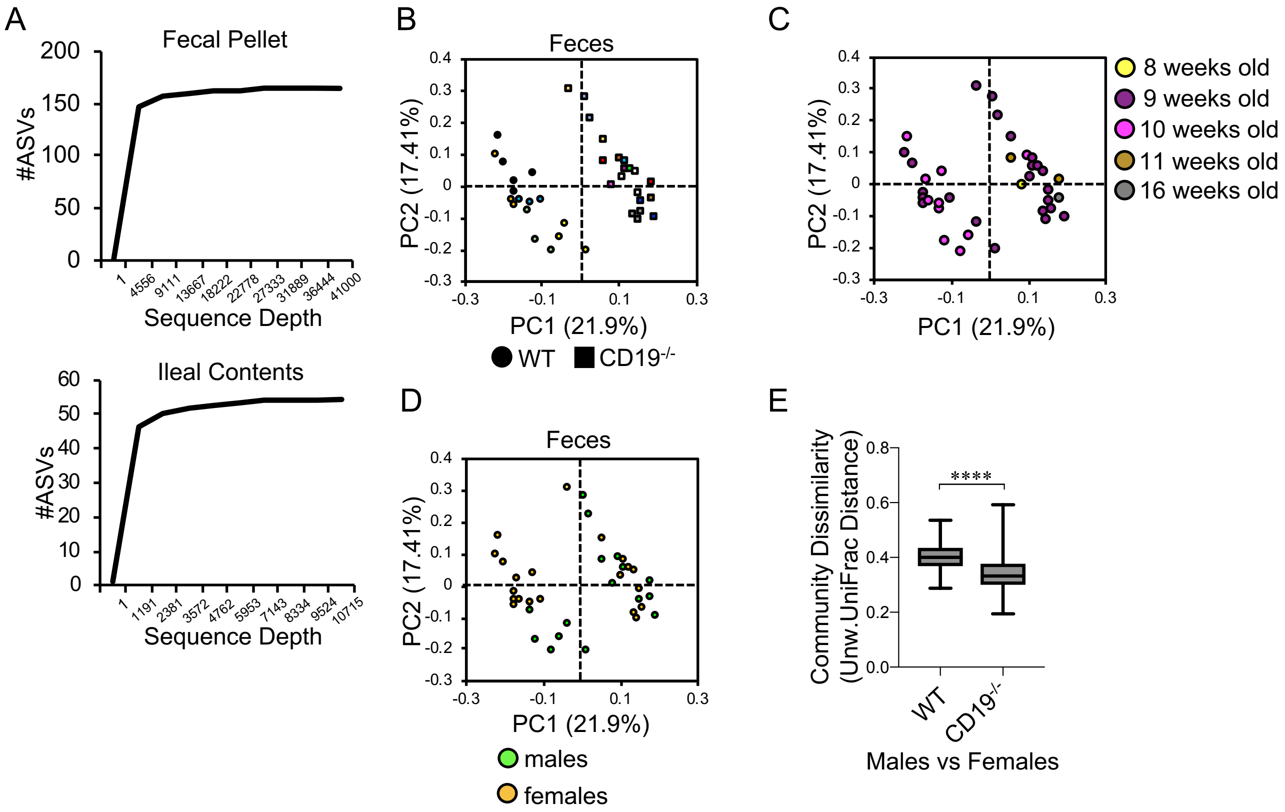

Supplementary Figure S1. Sampling for 16S microbiota profiling experiment and the effect of cage, age, and gender on microbiota composition. (A) Rarefaction plot of observed ASVs by sequence depth. (upper plot) Fecal samples were rarified to a depth of 41235 high-quality sequences per sample prior to analysis. (lower plot) Ileal samples were rarified to a depth of 10715 high-quality sequences per sample prior to analysis. **(B)** PcoA plot based on unweighted UniFrac analysis of β-diversity with samples colored by the cage animals were sampled from. Cage is not a significant driver of community divergence among individuals. **(C)** PcoA plot based on unweighted UniFrac analysis of β-diversity with samples colored by the age of animals used in analysis. **(D)** PcoA plot based on unweighted UniFrac analysis of β-diversity with samples colored by the gender of animals used in analysis. **(E)** Pair-wise comparison of community dissimilarity among male and female animals of each genotype. Student's t-test; ****=$p<0.0001$. Gender has a significant effect on community divergence in WT but not CD19$^{-/-}$ mice.

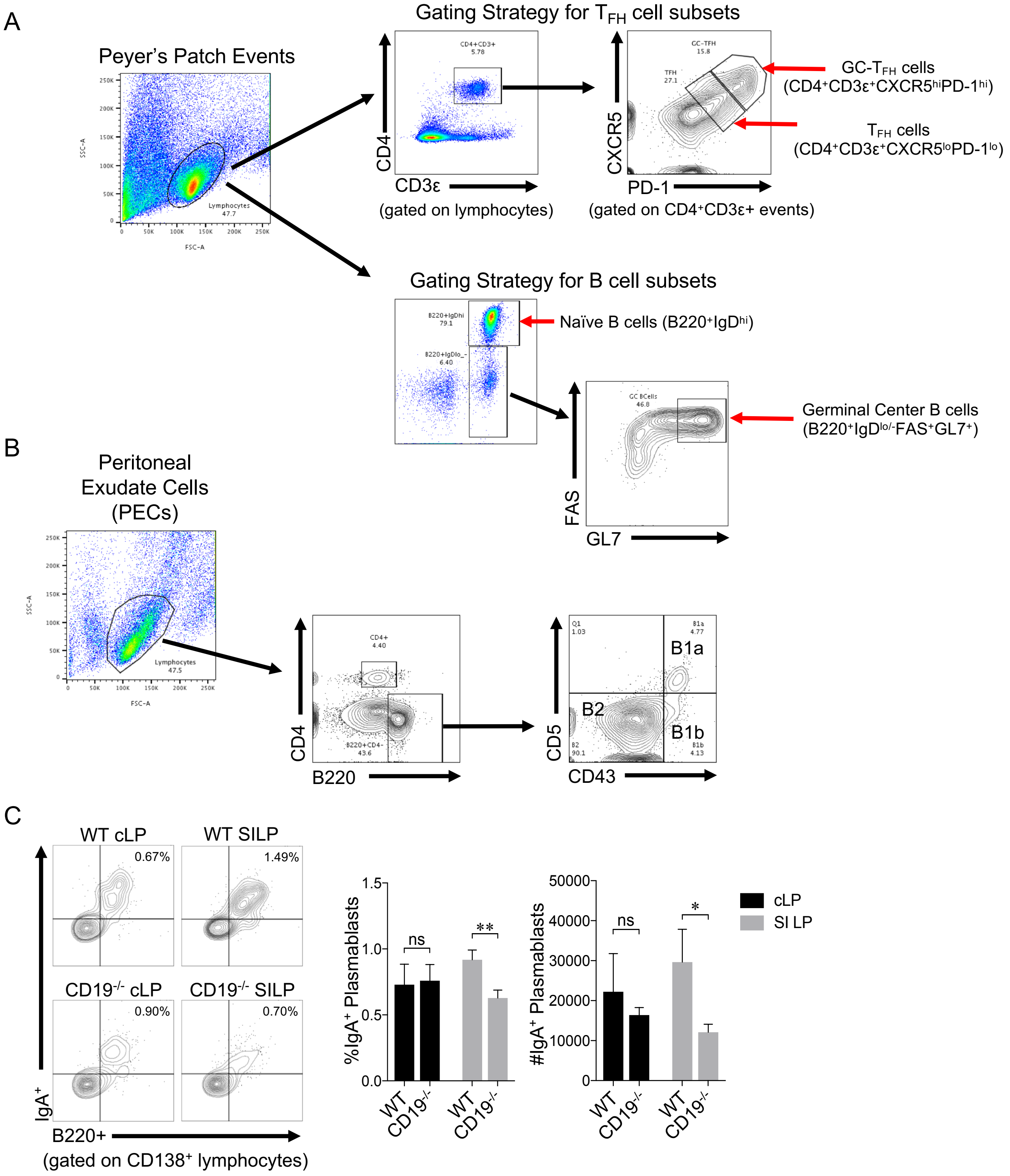

Supplementary Figure S2. Gating strategy for enumerating TFH and B cell subsets and comparison of the relative abundance of IgA⁺ plasmablasts in small intestinal and colonic lamina propria. (A) Representative flow cytometry plots demonstrating gating on T$_{FH}$ cell subsets and B cell subsets is shown. **(B)** Representative flow cytometry plots demonstrating gating on peritoneal B cell subsets is shown. **(C)** Representative flow cytometry plots of IgA⁺ plasmablast gating in lymphocytes isolated from the colonic lamina propria (cLP) and the small intestinal lamina propria (SI LP). The abundance of colonic and SI LP IgA⁺ plasmablasts was compared between WT (n=5) and CD19$^{-/-}$ (n=5) mice. Percentages shown reflect relative abundance of plasmablasts per total lymphocytes. (A, B) Student's t-test;*=$p<0.05$, **=$p<0.01$. Error bars represent S.D..

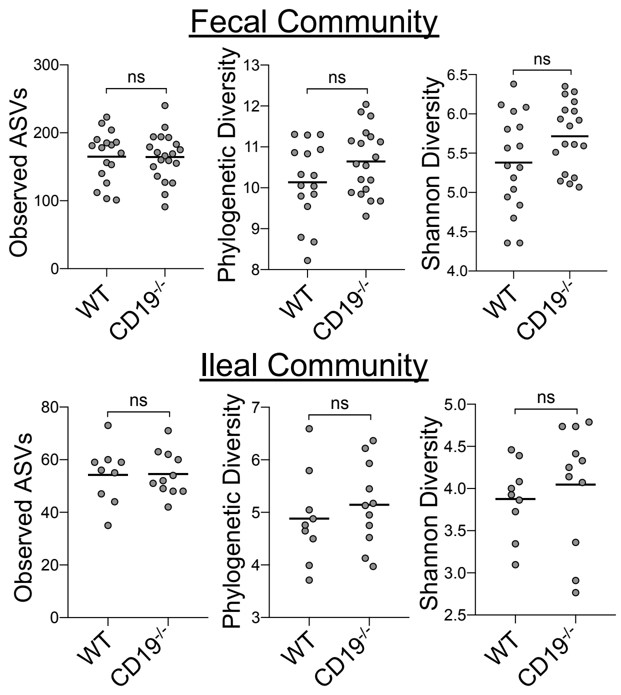

Supplementary Figure S3. Comparison of α-diversity between WT and CD19$^{-/-}$ microbiota communities. Pairwise comparison of α-diversity estimates (Observed ASVs, Faith's Phylogenetic Diversity, and Shannon Diversity) between genotypes are shown. Student's t-test; ns=not significant).

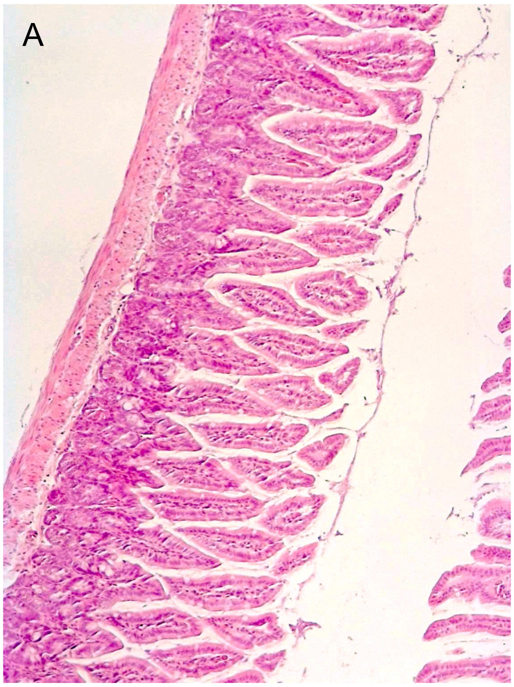

WT mouse ileum
(10X magnification)

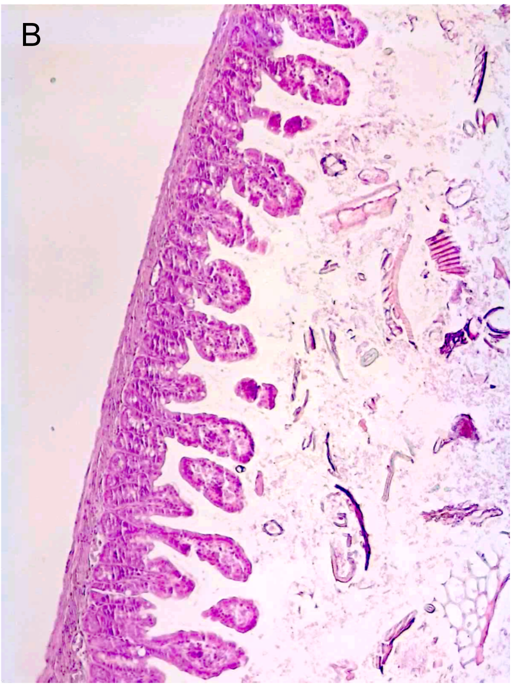

CD19$^{-/-}$ mouse ileum
(10X magnification)

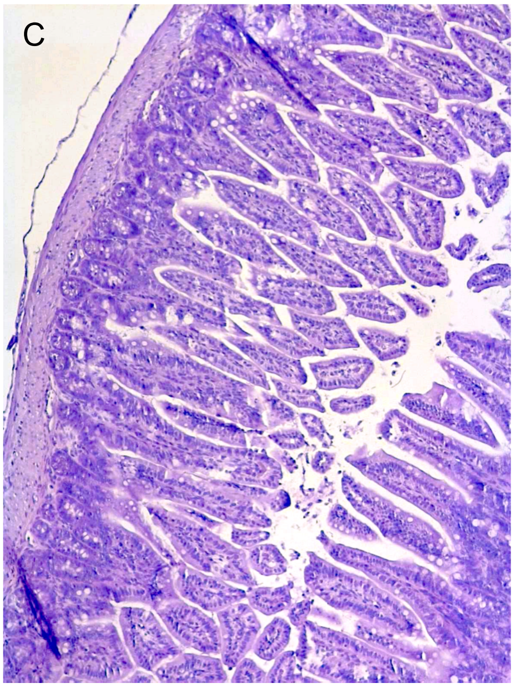

CD19$^{-/-}$ mouse ileum
(10X magnification)
(Metronidazole treated)

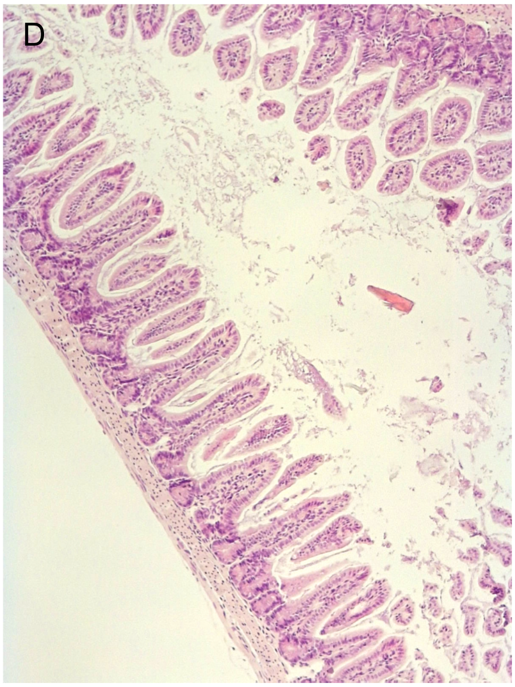

CD19$^{-/-}$ mouse ileum
(10X magnification)
(GFD treated)

Supplementary Figure S4. Representative histology sections of animal cohorts. High resolution images of H&E-stained ileal sections from **(A)** a WT mouse, **(B)** a CD19$^{-/-}$ mouse, **(C)** a CD19$^{-/-}$ moue treated with metronidazole, and **(D)** a CD19$^{-/-}$ mouse treated with GFD.

A

Scoring Rubric

Score=0: no inflammation observed

Score=1: low-grade inflammation, scattered mononuclear cell infiltration

Score=2: moderate inflammation, infiltrating mononuclear cells, lymphocytes, and plasmocytes

Score=3: high level of inflammation, transmural mononuclear cells, infiltrating lymphocytes and plasmocytes

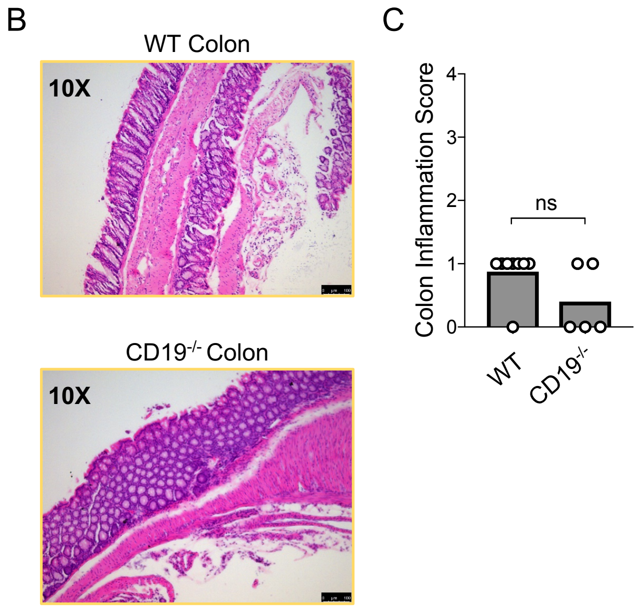

Supplementary Figure S5. Steady-state colonic inflammation is similar between WT and CD19$^{-/-}$ mice. (A) Scoring rubric followed by blinded pathologist to assess the degree of colonic inflammation. **(B)** Representative H&E stained longitudinal sections of colons from WT and CD19$^{-/-}$ mice. **(C)** Colonic inflammation scores of WT and CD19$^{-/-}$ mice during steady-state conditions. Students t-test; ns=non-significant.

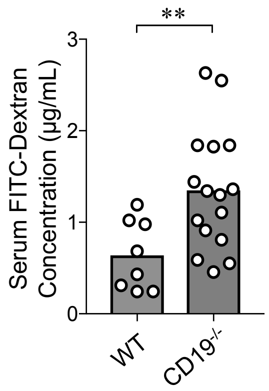

Supplementary Figure S6. Comparison of gut permeability between WT and CD19$^{-/-}$ mice under steady-state conditions. Fasted (8 hours) WT and CD19$^{-/-}$ mice were orally gavaged with 60mg/100g of FITC-dextran. Animals were euthanized, blood was drawn, and serum concentrations of FITC-dextran were measured on a fluorescent plate reader 4 hours later. Student's t-test; **=$p<0.01$.

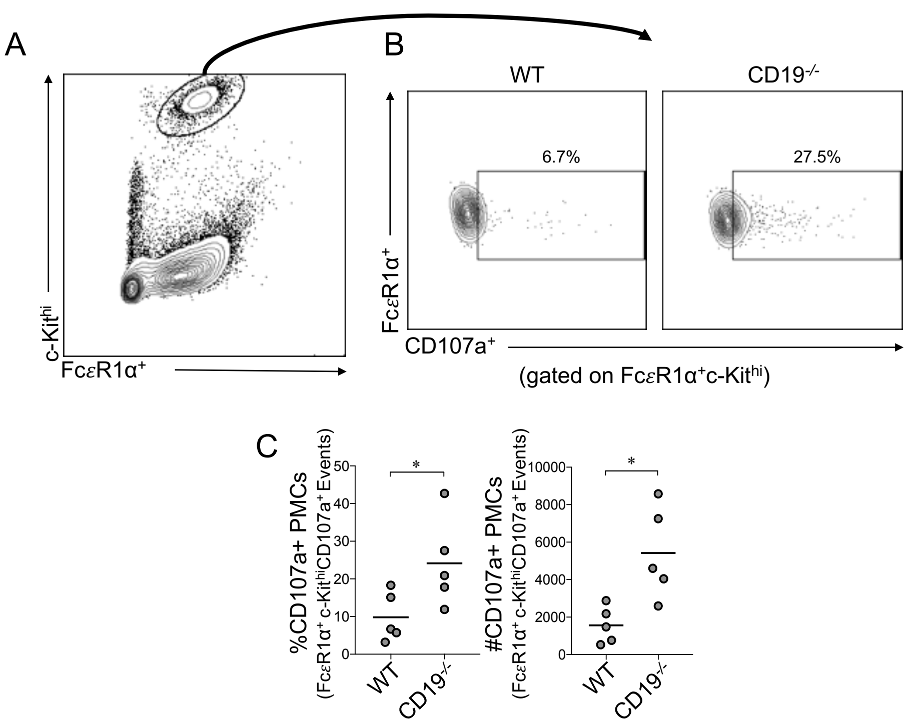

Supplementary Figure S7. Activated mast cells are enriched in the peritoneal cavity of CD19$^{-/-}$ mice. Peritoneal lavage fluid was collected from WT and CD19$^{-/-}$ mice and the abundance of mast cells (FCεR1α^{+}c-Kit^{hi}) were enumerated via flow cytometry. **(A)** Representative flow cytometry plot of gating strategy to identify mast cells in peritoneal fluid. **(B)** Representative flow cytometry plot to identify activated (CD107a^{+}) mast cells. **(C)** The relative and absolute abundance of mast cells in peritoneal fluids are shown. **(D)** The relative and absolute abundance of activated peritoneal mast cells are shown. Students t-test; *=p<0.05, **=p<0.01.
